# *CAMTA1* gene affects the ischemia-reperfusion injury by regulating *CCND1*

**DOI:** 10.3389/fncel.2022.868291

**Published:** 2022-09-09

**Authors:** Yang Liu, Guohui Shang, Xuran Zhang, Fuyong Liu, Chi Zhang, Zhihao Li, Jing Jia, Yan Xu, Zhaojing Zhang, Shangdong Yang, Baixue Zhou, Yingying Luan, Yanyang Huang, Yue Peng, Tianyi Han, Ying He, Hong Zheng

**Affiliations:** ^1^Department of Medical Genetics and Cell Biology, School of Basic Medical Sciences, Zhengzhou University, Zhengzhou, China; ^2^Department of Clinical Laboratory, The First Affiliated Hospital of Henan University of CM, Henan University of CM, Zhengzhou, China; ^3^Department of Pathogenic Biology and Immunology, School of Life Sciences, Sanquan College of Xinxiang Medical University, Xinxiang, China

**Keywords:** ischemic stroke, DNA methylation modification, *CAMTA1*, *CCND1*, cell cycle

## Abstract

Epigenetic modulations lead to changes in gene expression, including DNA methylation, histone modifications, and noncoding RNAs. In recent years, epigenetic modifications have been related to the pathogenesis of different types of cancer, cardiovascular disease, and other diseases. Emerging evidence indicates that DNA methylation could be associated with ischemic stroke (IS) and plays a role in pathological progression, but the underlying mechanism has not yet been fully understood. In this study, we used human methylation 850K BeadChip to analyze the differences in gene methylation status in the peripheral blood samples from two groups (3 IS patients vs. 3 healthy controls). According to their bioinformatics profiling, we found 278 genes with significantly different methylation levels. Seven genes with the most significant methylation modifications were validated in two expanded groups (100 IS patients vs. 100 healthy controls). The *CAMTA1* gene had significantly different methylation changes in patients compared to the controls. To understand the CAMTA1 function in stroke, we generated *CAMTA1* knockout in SH-SY5Y cells. RNA seq results in *CAMTA1* knockout cells revealed the pathways and gene set enrichments involved in cellular proliferation and cell cycle. Furthermore, a series of experiments demonstrated that in the oxygen-glucose deprivation/re-oxygenation (OGD/R) model system, the expression of cyclin D1, an essential regulator of cell cycle progression, was increased in SH-SY5Y *CAMTA1* KO cells. Increasing evidence demonstrated that ischemic stress could inappropriately raise cyclin D1 levels in mature neurons. However, the molecular signals leading to an increased cyclin D1 level are unclear. Our findings demonstrate for the first time that the *CAMTA1* gene could regulate cyclin D1 expression and implicate their role in strokes.

## Introduction

A stroke is an acute cerebrovascular incident. It affects the arteries leading to and within the brain, preventing oxygen and nutrients. About 80% of strokes are caused by ischemic compared to 20% hemorrhagic. It is one of the leading causes of death and disability worldwide. Currently, the stroke burden remains high, with 5.5 million deaths in 2016 based on the Global Burden of Disease report (Fogh et al., 2016). Besides, the stroke burden has also increased in young people aged 18–49 (GBD 2016 Stroke Collaborators, 2019). That implies massive public health issues and needs scaled-up prevention strategies. Therefore, a complete understanding of the pathogenesis of ischemic stroke (IS) is required.

Although substantial evidence has pointed to numerous environmental and genetic risk factors associated with IS (Giralt-Steinhauer et al., 2014; Feigin et al., 2017; GBD 2016 Stroke Collaborators, 2019; Singer et al., 2019), additional mechanisms remain clarified. Over the past years, with the improvement of research in the epigenetic field, multiple studies have revealed the epigenetic modifications involved in the pathogenesis of the cardiovascular disease (Traylor et al., 2012; Singer et al., 2019). One of the most understood epigenetic modifications, DNA methylation, usually occurs on the CpG site by adding one or more methyl groups to a cytosine. It affects gene transcription and expression without changing DNA sequences (Morgado-Pascual et al., 2018). Abnormal DNA methylation patterns have been investigated in IS pathogenesis (Feinberg, 2007; Udali et al., 2013). The global level of DNA methylation increased in the rat model of IS (Deng et al., 2019). Recently, more studies demonstrated that gene-specific methylation could also be involved in IS. For example, the hypermethylation in the cystathionine-beta-synthase promoter (Stanzione et al., 2020) and the AHCY gene encoding the S-adenosine homocysteine hydrolase (Dock et al., 2015) have been identified to increase ischemic stroke risk. People with hypomethylation in Long Interspersed Nucleotide Element-1 (LINE-1) may also have a higher risk of IS (Wang et al., 2019). Additionally, the higher methylation of cyclin-dependent kinase inhibitor 2B (CDKN2B) may affect arterial calcification in IS patients (Zhao et al., 2020). Despite these results, the new gene-specific methylation and its mechanisms remain unexplored.

Additionally, dysregulation of cell cycle machinery is also implicated in strokes. Empirical evidence suggests that inappropriately activated complex cyclin D1/cyclin-dependent kinases (Cdk) under ischemic stress conditions were responsible for the dysregulated cell cycle. The cyclin D1 levels were increased by the oxygen-glucose deprivation (Cai et al., 2009; Baccarelli et al., 2010b; Zhou et al., 2016). In addition, an increase in cyclin D1 immunoreactivity has also been detected in human stroke brains (Katchanov et al., 2001). However, the molecular signals leading to an increased cyclin D1 level have not been clarified.

In this study, we conducted a genome-wide analysis extracted from the peripheral blood to identify differentially methylated genes in IS patients using Illumina Infinium Methylation EPIC Bead Chip (850K chip) and Methyl Target (target regional methylation level sequencing). *CAMTA1* gene was the most highly methylated in patients compared to the controls. It encodes synthetic Calmodulin Binding Transcription Activator 1 (CAMTA1), which could inhibit the proliferation of various tumor cells, including breast cancer, colon cancer, pheochromocytoma, etc. Current research on the CAMTA1 protein has focused on its role in the pathogenesis of Epithelioid hemangioendothelioma (EHE), a malignant tumor of vascular endothelial cell origin. The primary mechanism of its pathogenesis is multiple translocations in the chromosome 1p36.3 and 3q25 regions, which happens to be the location of the *CAMTA1* gene. Long-term studies have found that *WWTR1-CAMTA1* gene fusion occurs in 90% of EHE cases. Under the transcriptional control of the WWTR1 promoter, this fusion gene activates the abnormally high expression of the *CAMTA1* gene, encoding a specific fusion transcription factor that plays a crucial role in the EHE pathogenesis. Few studies have reported that the *CAMTA1* gene was also associated with neurodegenerative diseases. Fogh et al. have shown that the *CAMTA1* gene affects the survival of patients with sporadic amyotrophic lateral sclerosis (ALS) (Fogh et al., 2016). Studies have shown that *CAMTA1* KO mice could develop ataxia, Purkinje fibrosis, and other characteristics (Long et al., 2014). However, the involvement of CAMTA1 in stroke has not been reported. Our results show that CAMTA1 knockout could attenuate oxygen-glucose deprivation/re-oxygenation (OGD/R)-induced apoptosis and block more cells at the S phase. Moreover, the *CCND1* mRNA and its coding protein: cyclin D1, were increased by decreased CAMTA1 levels. Our findings demonstrate that the *CAMTA1* gene affects the ischemia-reperfusion injury by regulating cyclin D1 proteins.

## Materials and methods

### Study population

The study was conducted on three healthy people and three acute cerebral infarction patients with magnetic resonance imaging scans performed in the Department of Neurology, the First Affiliated Hospital of the Henan University of Chinese Medicine, from May 2017 to September 2017. Two men and one woman with an average age of 57.3 years were in the IS group. Likewise, in the healthy control group, there are two men and one woman with an average age of 58.7 years. The validation of the population included 100 IS patients (60 men and 40 women), with an average age ranging between 58.34 and 72.1 years. The control group included 100 control groups (including 52 men and 48 women), with an average age ranging between 51.3 and 74.8 years.

Inclusion criteria: (1) The diagnosis of ischemic stroke conformed to the guideline for the diagnosis and treatment of acute ischemic stroke in China in 2010 and was confirmed by head MRI scans. (2) The course of the stroke was <1 week. (3) Over 18 years of age. Exclusion criteria: (1) Patients accompanied by unconsciousness, aphasia, or severe cognitive impairment could not cooperate with the examination. (2) Patients with psychosis or other psychiatric conditions such as anxiety, depression, and suicidal behavior. (3) Patients with other severe systemic diseases, including infection, cardiac and pulmonary failure, or hepatic and renal dysfunction. (4) Patients failed to perform MRI scans for various reasons. The Ethics Committee approved the study protocol of the First Affiliated Hospital of the Henan University of Chinese Medicine. All participants signed a written consent form.

Expanding clinical validation of population: peripheral blood samples were obtained from IS patients and stored at −80°C freezer. The inclusion criteria and exclusion criteria, as detailed above, were followed.

### Extraction of proteins

For mononuclear cell pellets, 100 μL of cell lysate buffer was added to the centrifuge tube (RIPA lysate is mixed well with PMSF in a 100:1 ratio) and readied for use. It was pipetted repeatedly until it was mixed well and the lysate was then transferred to a new 1.5 ml EP tube. It was incubated on ice for 30 min, during which time it was vortexed every 10 min to allow the cells to be fully lysed. The mixture was centrifuged at 12,000 rpm for 15 min at 4°C and the supernatant was transferred into a new 1.5 ml EP tube to obtain the total soluble protein from the mononuclear cells for subsequent experiments and stored at −80°C.

### DNA methylation

Peripheral blood samples from the six subjects were collected and used for the 850K DNA methylation analysis. The DNA microarray results were validated using the remaining samples.

After the frozen blood samples were thawed, the DNA from human whole blood cells was extracted with a Tiangen kit (Beijing, China, Catalog Number: DP319). DNA concentration and purity were quantified in a NanoDrop 2000 system (NanoDrop, Wilmington, DE). The DNA concentration must be higher than 50 ng/μl.

Whole DNA methylation (3 IS vs. 3 healthy controls) was assessed with the Infinium Human Methylation 850 BeadChip Kit (Illumina, Inc., San Diego, CA, United States), covering the human genome's 853,307 cytosine positions. Data preprocessing and analyses were conducted in the statistical programming environment R v3.1.2 with RnBeads v0.99. Normalization and background correction was applied to the methylation data with manufacture-recommended algorithms and implemented in the methylome package. Methylation levels were averaged for the replicates for each biopsy after normalization. We calculated the difference in methylation β-value between the two groups or the mean of the pairwise difference for paired samples. False discovery rates (FDR) were calculated using an improved Benjamini-Hochberg procedure to correct *p*-values for multiple hypothesis testing, and the methylation changes in CpG sites/regions with FDR < 0.05 were considered statistically significant. Ingenuity Pathway Analysis (IPA) was used to identify functional interactions of genes differentially methylated between groups. Average methylation signals on the CpG sites within each CpG site and/or promoter region were hierarchically clustered with Pearson dissimilarity and average linkage as clustering parameters.

### Screening of differential genes

Difference setting criteria: The absolute value of the Diff Score value between the case group and the control group sample was >13, and the Absolute value of Delta Beta was >0. 17, that is, the differential methylation gene.

Diff score = 10^*^sgn(13ref-13cond)4logl0(p) For a *P*-value of 0.05, Diff Score = 4–13

For a *P*-value of 0.01. Diff Score = 4–20

For a *P*-value of 0.001. Diff Score = 4–30.

The Ddta Beta value was calculated as the difference between the case group and the control group and Avg Beta was the degree of methylation difference between the case group and the control group at each site.

### Verifying candidate genes by methyl target region methylation sequencing

The methylation level of the promoter region of the *CAMTA1* gene in 100 IS cases vs. 100 healthy control samples was detected by methyl target region methylation sequencing. MethylTarget™ assays (targeted bisulfite sequencing) developed by Genesky Biotech (Shanghai, China) were carried out as previously described. Briefly, CpG sites adjacent to the promoter region of the *CAMTA1* gene were analyzed, and based on these CpG sites, four CpG regions from CpG sites in *CAMTA1* were sequenced (the relative distance from the transcriptional start site, amplification primers, and product size of these CpG regions are described in **Tables 2**, **3**). Genomic DNA was converted with bisulfite, and PCR was performed to amplify the targeted DNA sequences. The products were sequenced by an Illumina MiSeq benchtop sequencer (Illumina, CA, United States).

The total RNA was extracted using the Tiangen reagent (Beijing, China, Catalog Number: DP424). Using a QuantiTect Reverse Transcription kit (Vazyme, Wuhan, China, Catalog Number: R333-01), 2 μg of each RNA was reverse transcribed into cDNA. Expression levels of the genes were analyzed using a QuantiTect SYBR Green PCR kit (Vazyme, Nanjing, China, Catalog Number: Q221-01). The primer sequences are listed below:

Forward primer: gattatggtttgttttttaggatgagag
Reverse primer: aacccrattcaaactcrttcc.

### Western blot

Cells were lysed with RIPA lysis buffer and completed with protease inhibitor (Solarbio, Beijing, China, Catalog Number: R0020). Lysates were centrifuged at 4°C, 12,000 × g for 10 min, supernatants collected, and protein concentrations assessed using a BCA protein assay kit (Solarbio, Beijing, China, Catalog Number: PC0020). Equal amounts of protein were placed on 10% SDS-PAGE gels and blotted onto polyvinylidene difluoride membranes (Millipore, Hercules, CA, USA Catalog Number: IPVH00010). Membranes were blocked with 5% nonfat milk for 2 h at room temperature and then probed with CAMTA1(Abcam; Cambridge, UK, Catalog Number: ab251843), and cyclin D1 (ProteinTech, Wuhan, Hubei, China, Catalog Number: 60186-1-Ig) antibodies at 4°C overnight. The blots were then incubated with HRP conjugated secondary antibody. GAPDH and β-actin were used as an endogenous protein control. ECL substrates were used to visualize signals (ProteinTech, Wuhan, Hubei, China, Catalog Number: 60004-1-Ig).

### Bioinformatic analysis

For 850K chip and RNA seq omics results, we performed GO function annotation analysis based on the GO database (http://geneontology.org/page/go-database), and KEGG pathway annotation analysis based on the KEGG database (http://www.kegg.jp/kegg/ko.html).

### Cell culture and generation of *CAMTA1* knockout cell lines

Human neuroblastoma cell lines (SH-SY5Y) were maintained in Dulbecco's minimum essential medium (DMEM/F12, Seven biotech, Shanghai, China, Catalog Number: SC103-01) with 10% fetal bovine serum (FBS, ExCell Bio, Shanghai, China, Catalog Number: FSS500) and 1% penicillin/streptomycin (Gbico, MA, USA, Catalog 30-2220). All cells were cultured in an incubator with 5% CO_2_ at 37°C. Lipofectamine 3000 (Invitrogen, Shanghai, China, Catalog Number: L3000150) was used for miRNA transfection. Cells were assayed 48 h after transfection.

Knockout cell lines were generated using the CRISPR/Cas9 system. Cells were transfected with a px330 vector (Addgene, MA, USA, Catalog Number: 42230) encoding a gRNA for the gene of interest and a vector encoding a gRNA for the homo sapiens *CAMTA1* gene (5′-ggtatgtcgggaacctctcc-3′). Resistant clones were expanded after adding blasticidin selection (4 μg/ml).

To generate knockout HEK293T and SH-SY5Y cells, they were transfected with pLentiCRISPRv2 vector (Addgene, MA, USA, Catalog Number: 52961) encoding gRNAs targeting non-overlapping regions of the *CAMTA1* gene. Following puromycin selection (2 μg/ml, for 2 days), single cell clones were expanded, and gene disruptions were validated by sequencing.

The gRNA sequence CCGGGTCCTCCTCCGTAGTG was used to generate the SH-SY5Y *CAMTA1* knockout clone.

### siRNA transfection

*CCND*1 siRNA and non-sense siRNA (random siRNA) were purchased from Hanbio (Shanghai, China). The efficiency of transfection was evaluated using real-time RT-PCR. The sequences of *CCND1* and scrambled siRNAs are as follows: Target Sequence: CCACAGATGTGAAGTTCATTT, scrambled siRNA: CCGAAGTTACTATGAACAA. Transient transfection with siRNA was performed using Lipofectamine RNAiMAX reagent (Invitrogen), and siRNA was reverse transfected into cells according to the supplied protocol.

### Cell counting kit-8 assay and oxygen glucose deprivation/reoxygenation model

We applied cell counting kit-8 (Topscience, Shanghai, China, Catalog Number: TP1197) to assess cell proliferation. The cells were seeded on the 96-well plate with a density of 1,000 cells/well, 10 μL 5 mg/mL CCK-8 reagent was added to the well at 0, 24, 48, and 72 h. The culture was terminated 1 h after CCK- 8 regent adding, and the optical density OD value of each well was detected by a microplate reader (Tecan, Mannedorf, Switzerland) at 450 nm. The experiments were repeated in triplicate for each group.

OGD/R is the most common cell model in the study of ischemic stroke: 5 × 10^5^ cells were seeded in a 35 mm culture dish, and 2 ml of complete DMEM/F12 medium was added and placed in a regular cell incubator for a whole night. Further, 5 × 10^5^ cells were seeded in a 35 mm culture dish, 2 ml of complete DMEM medium was added and placed in a standard cell incubator for 4 h. Then cells were replaced with serum-free, dual-antibiotics and low-sugar DMEM medium (DMEM, sbjbio, Nanjing, China, Catalog Number: BC-M-038), and the cells were placed in an anaerobic incubator (HENGZI-HYQX-II, Shanghai, China) without O_2_ at 37°C for 4 h. (This step was to complete hypoglycemia and hypoxia and we checked cell viability at the corresponding time point).

The cells were taken out and replaced with a high-sugar complete medium and placed back into the regular cell incubator for several hours. (Reperfusion was completed in this step and cell viability was checked after 4 h).

### Colony formation assay

The cells were seeded on the 6-well plates with a density of 10,000 cells/well in triplicate in 3 ml of medium containing 10% FBS and allowed to grow for 3 days. The culture medium was replaced every day. After incubation, the medium was removed. The colonies were fixed with 4% paraformaldehyde for 15 min and then stained with hematoxylin for 15 min. The stained cells were rinsed three times with tap water to remove the excess dye. Each dish was then washed and dried. The colonies with a diameter larger than 0.6 mm were counted.

### Cell cycle assay

The cell cycle progression was assessed *via* a Cell Cycle Analysis kit (Beyotime, Shanghai, China, Beyotime, Shanghai, China, Catalog Number: C1052) in compliance with the manufacturer's instruction book. Then, cell proportion was measured at each phase through a flow cytometer (BD Biosciences, San Diego, CA, United States).

### TUNEL assay

According to the manufacturer's protocol, apoptotic DNA fragmentation was examined using the One Step TUNEL Apoptosis Assay kit (Beyotime Institute of Biotechnology, Haimen, China, Catalog Number: C1089). Briefly, cells were seeded into 24-well plates, respectively. Then, cells were fixed in 4% paraformaldehyde for 30 min at 4°C, permeabilized in 0.1% Triton X-100 for 2 min on ice, followed by the TUNEL assay for 1 h at 37°C. Cy3 (Cyanine 3)-labeled TUNEL-positive cells were imaged under a fluorescence microscope.

### Luciferase reporter gene

HEK293T *CAMTA1* KO cells (6 × 10^5^ cells/well) were cultured in 96-well plates and co-transfected with the control vector, *CAMTA1* overexpression vector, and the Renilla plasmid using Lipofectamine 3000 (Invitrogen, USA). The concentration of CAMTA1 overexpression vector is gradient-increasing from 0 to 100 ng. The procedure is performed according to the protocol of the kit (Catalog Number: MA0520-1). Firefly luciferase values were normalized to Renilla luciferase values, and the resulting ratios were used to express luciferase activities.

### Statistical analysis

We used SPSS 21.0 to perform statistical analysis. The distribution of variables was tested with the Kolmogorov Smirnov normal distribution test. Student's *t*-test or nonparametric test compared means of designated comparison groups.

## Results

### Characteristics of the patients included in the microarray analysis and validation

The population in Microarray: Total DNAs from the peripheral blood samples of three patients and three controls were extracted for the genomic DNA methylation assay. Table 1 presents the clinical characteristics of these six people (3 IS patients vs. 3 healthy controls). Compared to the control group, there were significant increases in TC, TG, and LDL levels and significant decreases in HDL levels in the IS group (*P* < 0.01), and no other significant differences were observed between these two groups.

**Table 1:** Characteristics of the patients included in the microarray analysis.

**Types**	**IS**	**Con**	***t*/χ^2^-value**	***P-*value**
	**(*n* = 3)**	**(*n* = 3)**		
Gender (male/female)	2/1	2/1	-	-
Age (years)	60.3 ± 9.5	65.6 ± 7.2	−0.986	0.38
Weight (kg)	62.5 ± 7.3	68.6 ± 8.0	0.976	0.385
MAP (mmHg)	95.1 ± 13.7	102.3 ± 15.2	0.609	0.575
BMI (kg/m^2^)	22.1 ± 4.3	24.9 ± 3.6	4.877	0.008
Total cholesterol (mmol/L)	7.55 ± 0.64	5.21 ± 0.53	0.929	0.405
Blood sugar (mmol/L)	6.930 ± 2.58	6.383 ± 2.89	0.245	0.819
Triglyceride (mmol/L)	3.581 ± 0.791	2.128 ± 0.169	3.111	0.036
HDL (mmol/L)	0.903 ± 0.252	1.422 ± 0.194	2.827	0.048
LDL (mmol/L)	3.834 ± 0.960	2.101 ± 0.683	2.938	0.042
HbA1c (%)	6.58 ± 1.74	6.13 ± 1.95	0.298	0.780

MAP, mean artery pressure; BMI, body mass index; LDL, low density lipoprotein; HDL, high density lipoprotein.

The population of validation: Total DNA from the peripheral blood samples of 100 patients and 100 controls was extracted for the blood genomic DNA methylation assay. Table 2 lists the clinical characteristics of these 200 people (100 IS patients and 100 controls). TC, TG, LDL, and HDL levels were significantly different between these two groups. More details of these two groups can be seen in Supplementary Tables 1, 2.

**Table 2:** Characteristics of the patients of the verification population.

**Types**	**(*n* = 100)**	**(*n* = 100)**	***t*/χ^2^-value**	***P-*value**
Gender (male/female)	55/45	58/42	0.183	0.669
Age (years)	61.3 ± 8.5	67.4 ± 7.8	0.083	0.934
Weight (kg)	69.5 ± 7.6	67.6 ± 8.5	1.82	0.07
MAP (mmHg)	102.3 ± 16.6	96.3 ± 12.8	2.858	0.005
BMI (kg/m^2^)	23.3 ± 4.6	22.6 ± 4.3	1.112	0.268
Total cholesterol (mmol/L)	4.73 ± 0.55	5.50 ± 1.51	4.791	0.000
Blood sugar (mmol/L)	6.59 ± 2.58	6.17 ± 2.36	1.201	0.231
Triglyceride (mmol/L)	2.881 ± 0.531	3.958 ± 0.459	15.334	0.000
Albumin (g/L)	40.63 ± 4.73	39.38 ± 5.53	1.718	0.087
HDL (mmol/L)	1.243 ± 0.252	0.979 ± 0.194	8.301	0.000
LDL (mmol/L)	2.689 ± 0.570	3.156 ± 0.669	5.313	0.000

MAP, mean artery pressure; BMI, body mass index; LDL, low density lipoprotein; HDL, high density lipoprotein.

### Global changes in blood genomic methylation patterns in IS

**Figure 1:**
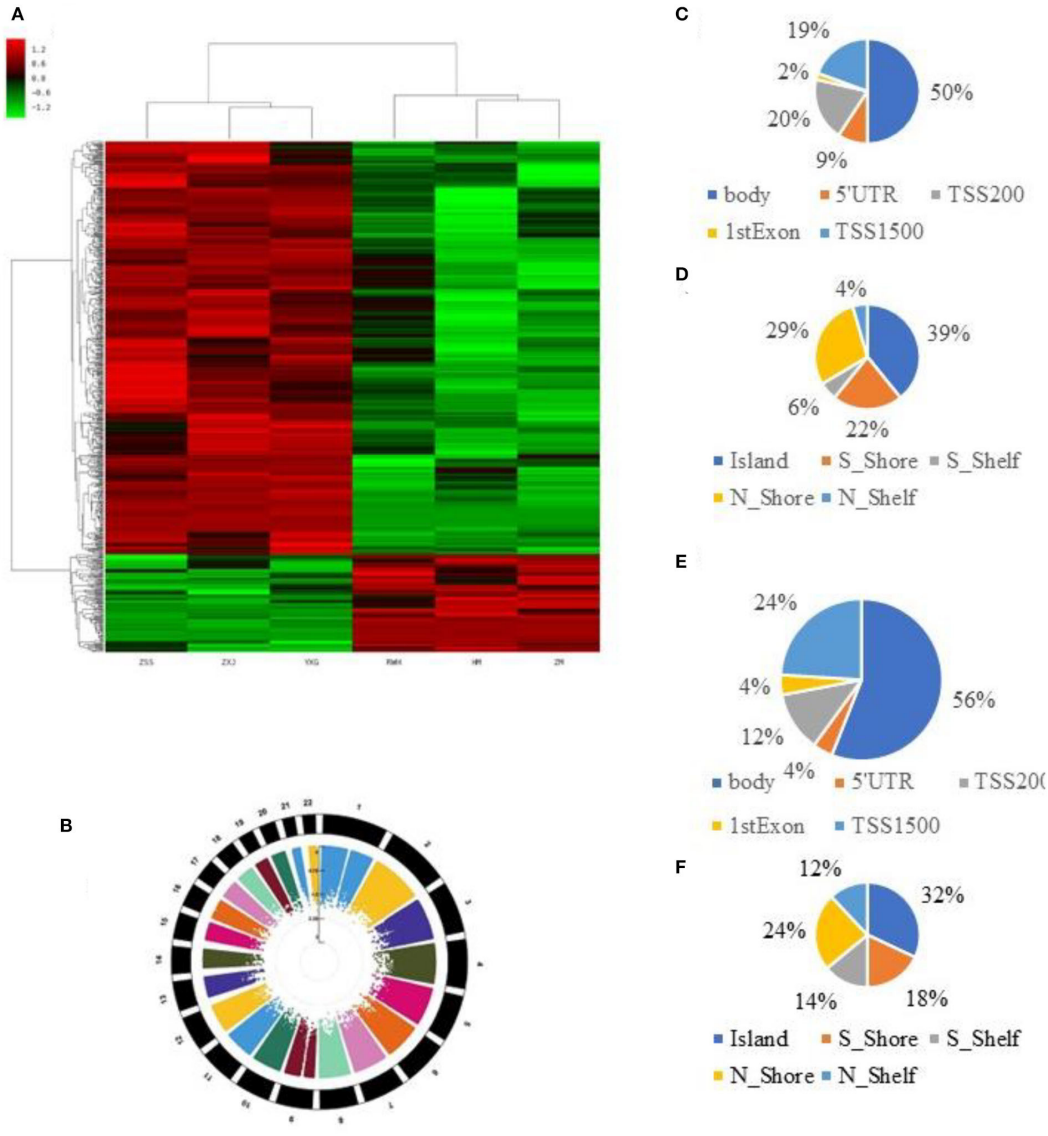
850K core piece difference in step base point result. **(A)** Heat map showing methylation level (β-value) in cases and controls. **(B)** Multitrack Rectangular-Manhattan plot in cases and controls. **(C)** The distribution of DMCs according to the site regions of the hypomethylation. **(D)** The distribution of DMCs according to the genomic regions of the hypomethylation. **(E)** The distribution of DMCs according to the site regions of the hypermethylation. **(F)** The distribution of DMCs according to the genomic regions of the hypermethylation.

We used Genome Studio V2018 software to report the β-values of 853,307 DNA methylation sites for the samples from the controls (*n* = 3) and the IS patients (*n* = 3) (Figure 1A). Statistical analysis revealed that 622 sites showed a difference in the degree of methylation; 502 sites were hypermethylated, and 120 sites were hypomethylated, with a ratio of 4:12. Manhattan map shows the distribution of methylation sites on chromosomes (Figure 1B).

Most hypomethylated sites were located on chromosomes 2, 5, 6, and 7, the most hypermethylated sites were on chromosomes 1, 2, and 6, and the most overall differentially methylated sites were located on chromosomes 1 and 6. The distribution of CpG sites in different regions of genes is shown in Supplementary Figures 2A,B.

Next, we carried out an analysis according to the functional domains of DNA. Two hundred and fifty-one sites (50%) situated within 1,500 bp upstream of the transcription start site (TSS 1500) among the hypermethylated sites, followed by 100 sites situated within 200 bp upstream of the transcription start site (TSS 200). Ninety-five sites (19%) were located at the gene bodies. In addition, the smallest percentage (0.02%) was in the 3′UTR for hypermethylated loci, while mostly hypomethylated loci had the smallest percentage in the first exon, 5′UTR and 3′UTR (0.04%) (Figure 1C).

Among the hypomethylated sites, 120 sites were located at gene bodies (50.0%), followed by 40 sites situated within 1,500 bp upstream of TSS (19.4%) and 40 sites at noncoding intergenic domains (19.4%) (Figure 1D).

When comparing the IS group to the control group, we observed that most of the hypermethylated loci (42.59%) were found in CpG sites, while most of the hypomethylated loci were in the open sea (70.57%) (Figure 1E). The smallest percentage (4.33%) of hypomethylated loci was in sites, compared to the smallest percentage of hypermethylated sites (3.06%) found on shores (Figure 1F). The distribution of DMCs according to the island regions of the hypomethylation and hypomethylation is shown in Supplementary Figures 1A,B.

### Bioinformatics analysis of differentially methylated genes of whole blood from IS patients

The differentially methylated sites were analyzed concerning known functional genes with the DAVID bioinformatics database. Among the 622 differentially methylated sites, the top 19 genes with the greatest extent of hypermethylation were *ABCA1, ADAMTSL5, COLgA2, ERCC5, TGFB1, ABCG1, ATP10A, CYP2E1, HOX4A, PCDHB7, ARL4C, SULF2, KLF11, ADARB2, GDFl5, CDHJ5, CAMTA1, SMG6*, and *RNFl44b* (Supplementary Figure 3). After analyzing the methylation detection data of the target region, especially the methylation level of the differential site, the methylation levels of the 19 candidate genes in different CpG sites are described in Table 3. *ABCA1, ADARB2, ATPI0A, CAMTA1, CDHI5, COL942*, and *TGFB1* have differences in the overall methylation level of CpG sites between the IS group and the healthy control group, and they are statistically significant (at the same time satisfying the triple test, that is, *T*-test *P-*value <0.05, *U*-test *P*-value < 0.05 and logistic regression analysis *P*-value < 0.05). Among the above seven genes (marketed ^*^ in Table 3), the CpG site in the *CAMTA1* promoter region was hypermethylated in the IS case group. The CpG site of the promoter region of six genes, *ABCA1, ADARB2, ATPI0A, CDHI5, COL942*, and *TGFβ1*, was hypomethylated in the IS case group.

**Table 3:** Genes with significant differences in CpG site methylation levels.

**Genes**	***P-*value (*T*-test)**	***R-*value (*U*-test)**	***R-*value (logistic)**	**OR (L95-U95) (logistic)**	**Methyl diff**
*ABCA1_1*	0.1168	0.0876	0.1237	0.1100 (0.0066–1.8294)	−0.00121
ABCA1_2^*^	0.0050*	0.0032*	0.0102*	0.1234 (0.0246–0.6191)	−0.00425
*ABCG1_1*	0.3874	0.3107	0.3769	1.2366 (0.7720–1.9806)	0.0045
*ABCG1_2*	0.7507	0.4457	0.7444	13221 (0.2467–7.0848)	0.00037
*4DAMTSL5J*	0.0725	0.0682	0.0825	1.9938 (0.9149–4.3449)	0.00586
*ADA/fTSL5_2*	0.1073	0.0391	0.1183	5.6142 (0.644148–0.9353)	0.00165
ADARB_1*	0.0161*	0.0328*	0.0299*	0.0982 (0.0121–0.7982)	−0.00316
*ADARB2_2*	0.1428	0.0696	0.1456	0.4684 (0.686–1.3011)	−0.00301
*ARL4C_1*	0.7746	0.7473	0.7679	1.8657 (0.0296–117.429)	0.00013
*ARI,4C_2*	0.1067	0.0732	0.1160	3.6092 (0.728247–0.8893)	0.00221
ATPIOA_1*	0.0264*	0.0045*	0.0415*	0.0612 (0.0042–0.8992)	−0.00234
CAKfTAl1_1*	0.0286*	0.0348	0.Q387*	247764.8000 (1.9042–32 238134221.0000)	0.00042
*CAMTAI_2*	0.4899	0.2708	0.4799	0.0512 2 (0.0000–195.381)	−0.00017
*CAMTAI_3*	0.6222	0.8309	0.6109	0.4530 (0.0214–9.5705)	−0.00037
*CAMTA1_4*	0.7710	0.8287	0.7638	0.9715 (0.8043–1.1734)	−0.0033
CDH15_1*	0.0408	0.0102	0.045 P	0.8533 (0.7257–1.0035)	−0.03357
COL9A21^*^	0.0151*	0.0589	0.0253*	0.0046 (0.0000–0.5159)	−0.00125
*COL9A2_2*	*0.5745*	0.8063	0.5655	0.8485 (0.4844–1.4861)	−0.00201
*CYP2E1_1*	0.1898	0.1760	0.1859	0.9616 (0.9074–1.0190)	−0.04958
*CYP2E1_2*	0.4219	0.4773	0.4116	0.9807 (0.9360–1.0275)	−0.03444
*ERCC5_1*	0.9926	0.3862	0.9925	0.9914 (0.1607–6.1143)	−0.0000098
*ERCC5_2*	0.4109	0.5268	0.4012	5.0355 (0.1155–219.515)	0.00043
*GDF15_1*	0.5398	0.4304	0.5298	1.0709 (0.8649–1.3259)	0.00572
*H0XA4_1*	0.1381	0.1258	0.1570	1.0787 (0.9713–1.1980)	0.07174
*HOXA4_2*	0.0606	0.0927	0.0679	7.0837 (0.8654–57.9837)	0.00192
*KLFU_1*	0.7047	0.6891	0.7038	1.0456 (0.8308–13160)	0.00333
*KLFU_2*	0.4291	0.6146	0.4208	0.9219 (0.7563–1.1238)	−0.00810
*KLFU_1*	0.0952	0.1989	0.1045	1.0586 (0.9882–1.1340)	0.05322
*PCDHB7_1*	3,606	0.3862	0.3534	0.9594 (0.8789–1.0472)	−0.02123
*PCDHB7_1*	0.3076	0.4006	0.2946	0.9154 (0.7758–1.0800)	−0.01267
*RNF14-4B_1*	0.0551	0.0451	0.0744	0.0263 (0.0005–1.4318)	−0.00119
*SMG6_1*	0.8594	0.7864	0.8550	0.5022 (0.0003–812.876)	−0.000047
*SULF2_1*	0.8628	0.8264	0.8586	0.8899 (0.2465–3.2125)	−0.00027
*SULF2_2*	0.1106	0.0980	0.1169	0.5068 (0.2167–1.1854)	−0.00406
*SULF2_1*	0.3171	0.0927	3,169	0.6329 (0.2583–1.5505)	−0.00238
TGFβ1*	0.00/2*	0.0006*	0.0052*	0.5052 (031270–0.863)	−0.01798

“OR(L95-U95) (Logistic)” means the OR value of the regression regression and 95% confidence interval: “MethylDif” means the degree of difference in methylation between the two groups-the average degree of methylation in the case group-the control group Average degree of methylation.

**Figure 2:**
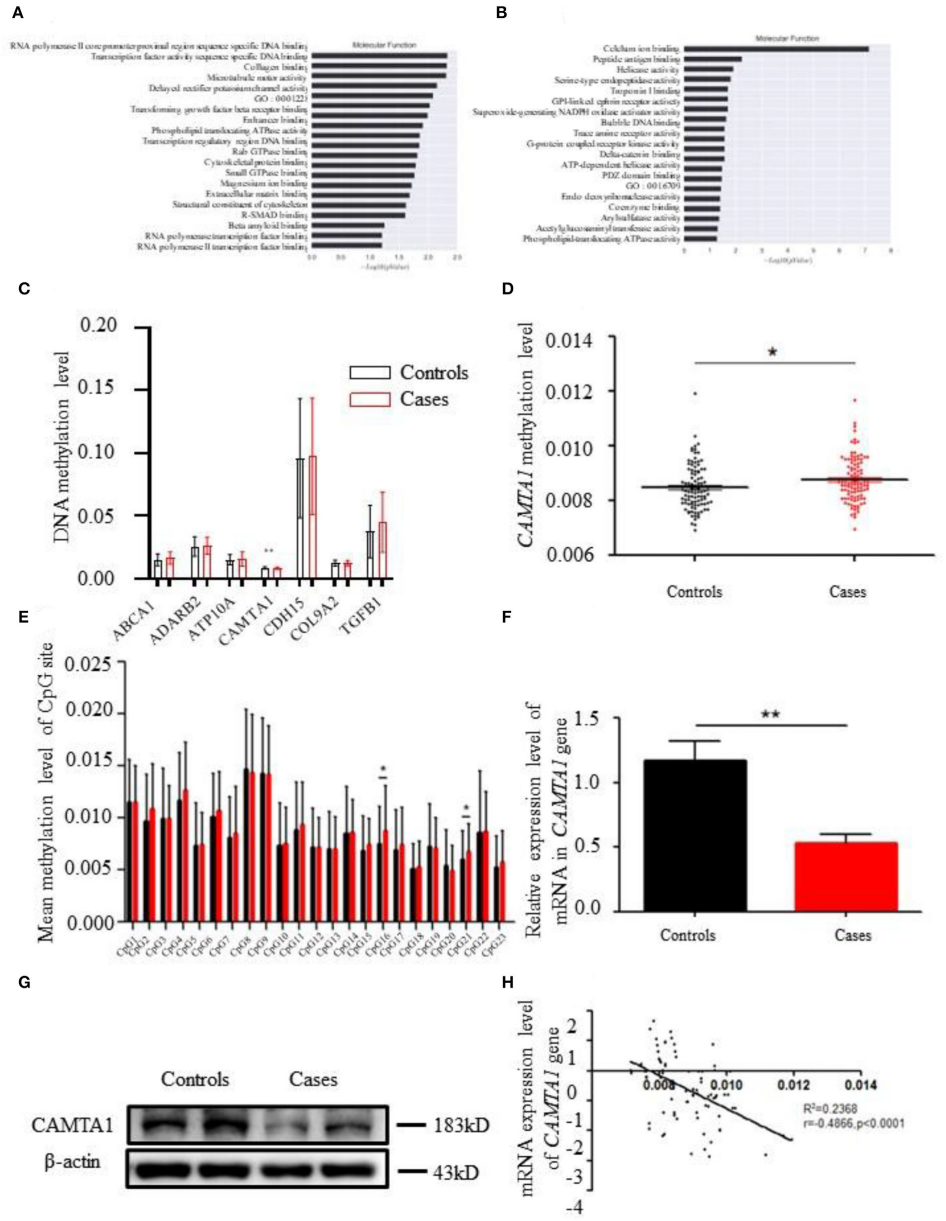
The bioinformatics results of 850K methylation microarray. **(A,B)** KEGG analysis results of different genes in cases and controls. **(C)** Comparison of DNA methylation differences of seven genes. **(D)** Methylation levels of *CAMTA1* gene in cases and controls (*P < 0.05). **(E)** Comparison of the average methylation levels of 23 CpG sites in the *CAMTA1* gene promoter region between two groups (*P < 0.05). **(F)** *CAMTA1* mRNA expression in the expanded population (**P < 0.01). **(G)** CAMTA1 protein expression in patients and healthy people. **(H)** Correlation between *CAMTA1* gene expression level and methylation level.

We undertook the gene ontology and pathway analysis of 502 differentially methylated sites to categorize them according to their biological functions and pathways. As Figures 2A,B show, the KEGG pathway analysis affiliated them with numerous pathways. The highest number of genes participated in Cell Adhesion Molecules (CAMs) (142 genes, *P* = 0.0021). Morphine addiction and the Hippo signaling pathway were involved in 92 (*P* = 0.028) and 154 (*P* = 0.042) genes, respectively. The genes hypermethylated CpG mapped were involved in the Hippo signaling pathway (154 genes, *P* = 0.014), Dorso-ventral axis formation (27 genes, *P* = 0.028), and Arrhythmogenic Right Ventricular Cardiomyopathy (ARVC) (74 genes, *P* = 0.032). The hypomethylated genes were implicated in CAMs (142 genes, *P* = 0.0008), Allograft rejection (37 genes, *P* = 0.005), and Graft-vs.-host disease (41 genes, *P* = 0.007) (**Figures 4D,E**; Supplementary Tables 4, 5).

The GO pathway analysis results are presented in Supplementary Tables 6–8. GO enrichment analysis in the categories of cellular component (CC) revealed that 10 GO terms from CC were significantly enriched in patients (Figure 2D). The top three terms are listed as follows: (1) early endosome membrane; (2) MHC class I protein complex; (3) cell junction. Furthermore, homophilic cell adhesion *via* plasma membrane adhesion molecules, cell adhesion, and phospholipid translocation were the top three pathways in biological processing systems (BP pathways). The differentially methylated genes were arranged into 10 groups based on their molecular function (e.g., biochemical cascade) like calcium ion binding, phospholipid-translocating ATPase activity, and collagen binding.

### Validation of the differentially methylated CpG Loci in *CAMTA1*

After analyzing the methylation detection data of the target region, especially the methylation level of the differential site, the methylation levels of the 19 candidate genes in different CpG sites were validated. *ABCA1, ADARB1, ATP10A, CAMTA1, CDH15, COL9A2*, and *TGFβ1* were the top seven genes having significantly different methylation levels (Figure 2C). The *CAMTA1* gene presents the most different hypermethylation levels between the two groups (Figure 2D).

Next, we explored the most common methylation sites of *CAMTA1*, located on Chr1. We screened out a CpG site in the promoter region of the *CAMTA1* gene through website prediction. The sequence of this region is as follows, with a total of 23 CpG sites, marked in red: GACTCATGGTTGCCTGTCCCAGGATGAGGCCGCCGGCCGAAAGCAGAGAAGCGCCAGCCCCGGCCGCCCGGGTGGAGCGCTGGGCAGCCGAGTTTCCCACCCTCCTCAATCCGGAGAGTCCGCGCGGGGCTTTTCCTAATAAATAGCCAGGCACCCGCTGCCCTCGCGCTGCGTACGGGAGCACGTGCCCCCCGGGAGGTGGGCGCCCGCCAGGTGCCCGAACGAGCCTAGGAACCGGGTCGGGAACGAGCCTAGGAACCGGGTC.

We compared mean methylation levels at 23 CpG sites in the promoter region of the *CAMTA1* gene in the control and case groups. In the *CAMTA1* display, 16 CpG and 21 CpG sites showed a significant difference (*P* < 0.05; Figure 2E). We found elevated methylation levels in the promoter region of the *CAMTA1* gene in ischemic stroke patients, at the same time with decreased *CAMTA1* mRNA expression (Figure 2F). As a consequence, its protein level was also reduced in IS patients (Figure 2G). To validate the correlation of DNA methylation with gene expression, we analyzed the Pearson correlation between the degree of methylation in the promoter region of the *CAMTA1* gene and its corresponding *CAMTA1* mRNA expression level (*P* < 0.0001; Figure 2H).

### *CAMTA1* knockout accelerated cell proliferation and inhibited apoptosis

**Figure 3:**
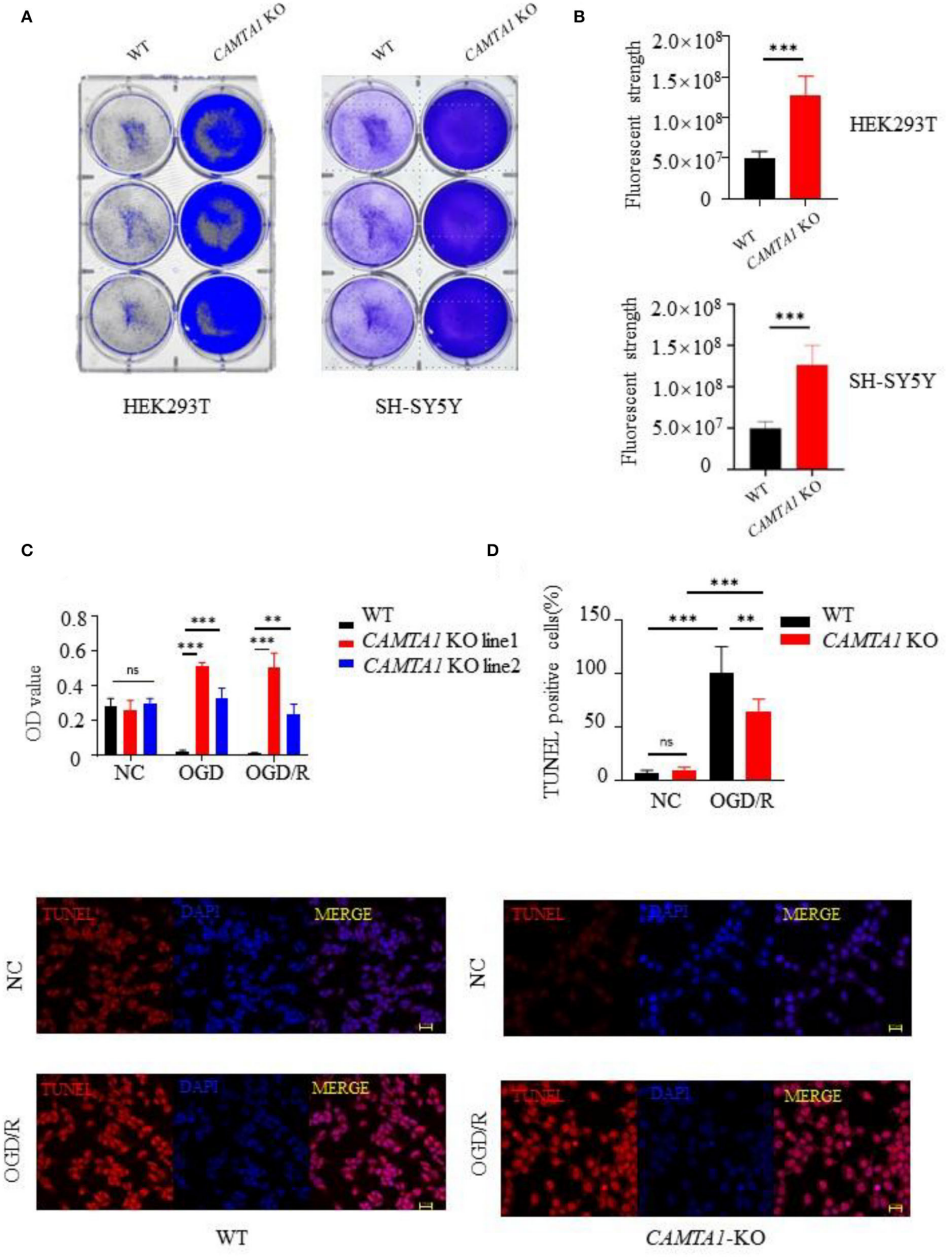
*CAMTA1* KO influences cell proliferation. **(A,B)** Gentian Violet staining shows differences in cell proliferation in HEK293T and SH-SY5Y cell lines (**P < 0.01, ***P < 0.001). **(C)** Relative viabilities of HEK293T and SH-SY5Y cells after incubation with OGD/R treatments (**P < 0.01). **(D)** Cell apoptosis was detected by TUNEL staining (*P < 0.05, **P < 0.01, ***P < 0.001). ns stand for not statistically different.

To investigate the CAMTA1 function implicated in stroke, we did knockout *CAMTA1* gene in HEK 293T and SH-SY5Y cell lines by Crisper/Cas9 (Supplementary Figure 1D). The qPCR results showed that the mRNA of *CAMTA1* had decreased successfully (Supplementary Figure 1E). The HEK 293T cells are a well-established tool. Moreover, the SH-SY5Y cell line was selected because it has a higher expression of *CAMTA1* mRNA compared to other neural cells (Supplementary Figure 1C). The staining of cells with crystal violet dye showed that the number of staining cells significantly increased in both *CAMTA1*-KO cells (Figures 3A,B) due to the accelerated cell proliferation (Figure 3C). Furthermore, the TUNEL assay demonstrated that decreased *CAMTA1* protected cells from oxygen-glucose deprivation/reperfusion (OGD/R) injury, and the TUNEL positive cells decreased in *CAMTA1*-KO cells compared after OGD/R (Figure 3D). Taken together, these results indicate that decreased *CAMTA1* levels could influence OGD/R injury.

### The transcriptomic study found that *CCND1* was upregulated in SH-SY5Y *CAMTA1*-KO cell line

Next, we used the RNA-seq approach to analyze the transcriptomes of two cell lines :SH-SY5Y and HEK293T. The genes having different expression levels in two cell lines are shown in Supplementary Table 9. We performed GO term and KEGG pathway enrichment analysis to analyze this signature further. From the KEGG enrichment results, it listed the top six KEGG pathways of the hypermethylated genes: focal adhesion (31 genes, *P* = 0.0076), Hippo signaling pathway (26 genes, *P* = 0.0076), cellular senescence (25 genes, *P* = 0.0121), p53 signaling pathway (15 genes, *P* = 0.0202), chronic myeloid leukemia (15 genes, *P* = 0.0308) and ECM-receptor interaction (14 genes, *P* = 0.0446). The pathways of hypomethylated genes are Ribosome (94 genes, *P* = 0.0076), Huntington's disease (81 genes, *P* < 0.0001), Parkinson's disease (63 genes, *P* < 0.0001), Oxidative phosphorylation (60 genes, *P* < 0.0001), RNA transport (63 genes, *P* < 0.0001), and Spliceosome (53 genes, *P* < 0.0001).

**Figure 4:**
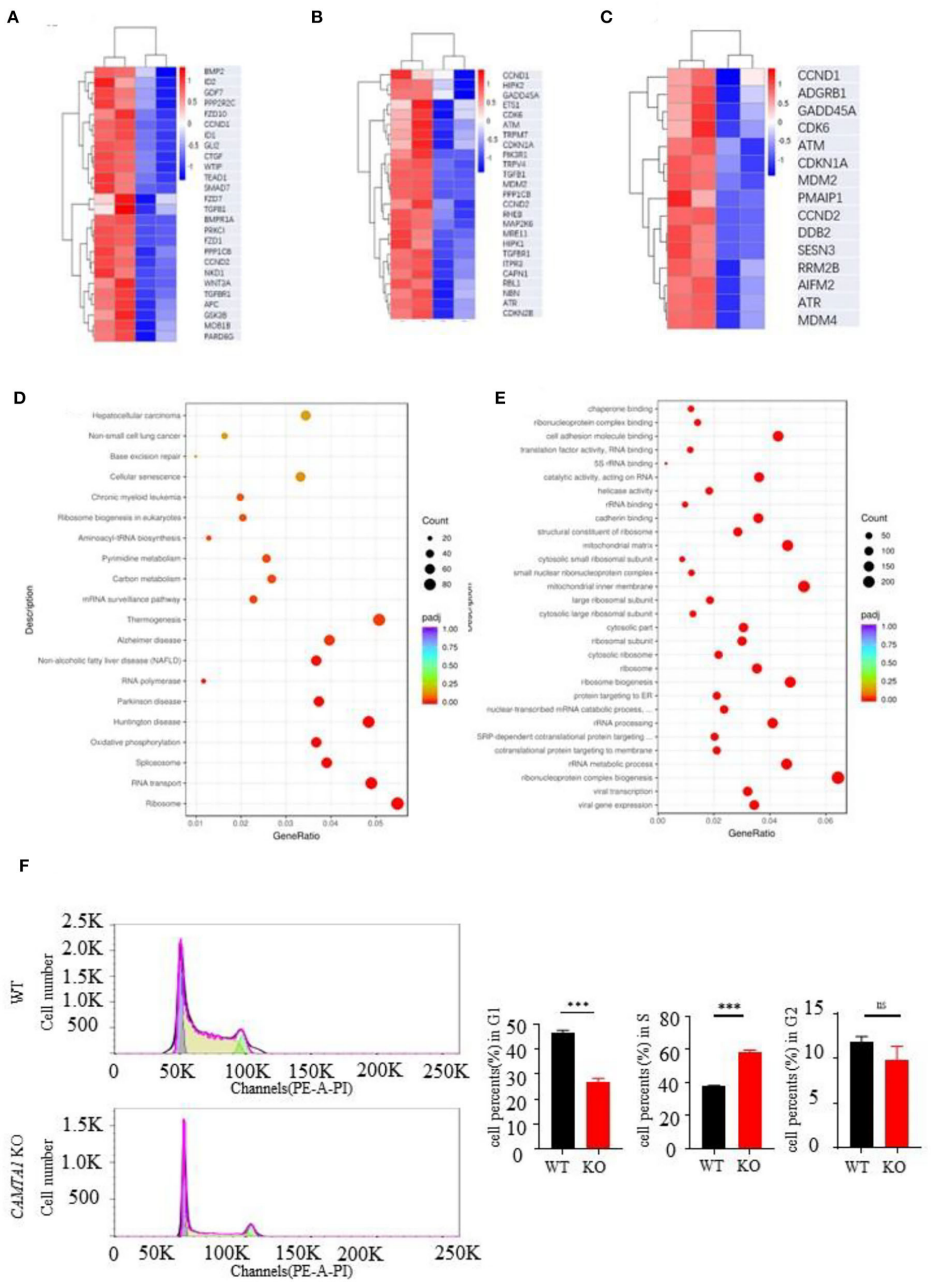
RNA seq results of *CAMTA1* KO SH-SY5Y cell lines and WT cell lines. **(A–C)** Heatmap of P53 relative genes, senescence genes, and Hippo pathways of the RNA seq in *CAMTA1* KO SH-SY5Y cell lines. **(D)** Bubble plot shows the significant GO pathways involved by the *CAMTA1* KO SH-SY5Y cell lines. **(E)** Bubble plot shows the significant KEGG pathways involved by the *CAMTA1* KO SH-SY5Y cell lines. **(F)** Flow cytometry histograms of actively dividing and quiescent cells. The percent of cells in each cell cycle phase is shown above the peaks (**P < 0.01, ***P < 0.001). ns stand for not statistically different.

The GO analysis results are shown in the Supplementary materials. We expected to find some pathways to explain the excessive proliferation of the *CAMTA1* KO cells. The heat map of the genes involved in the Hippo signaling pathway, cellular senescence pathways, and p53 signaling pathway is presented in Figures 4A–C. The heat map of the whole genes is shown in Figures 4D,E. These results revealed that the *CCND1* gene was upregulated and implicated in all these pathways. Interestingly, the cell cycle analysis by flow cytometry (Figure 4F) demonstrated that more SH-SY5Y *CAMTA1*-KO cells entered the S phase. As we know, the *CCND1* gene codes cyclin D1 is an important cell cycle regulator that controls the transition from G1 to the S phase. Meanwhile, many studies in mouse models and different neural cell lines demonstrated that cyclin D1 level was increased under ischemic stress conditions. Therefore, we suppose that CAMTA1 could regulate cyclin D1, and through this intermediate CAMTA1 could play a role in stroke.

### CAMTA1 may regulate cyclin D1 to control the cell cycle

**Figure 5:**
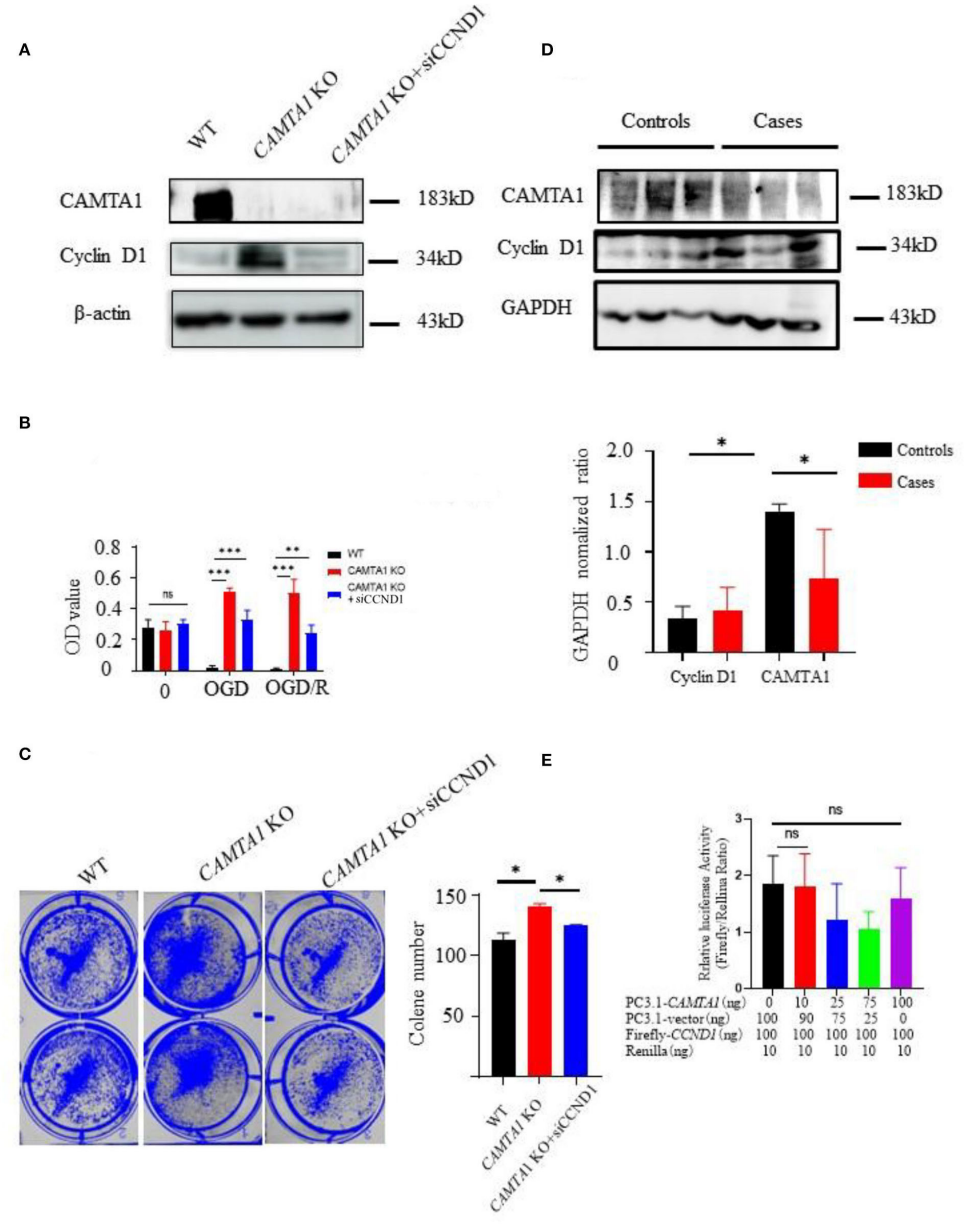
The downregulation of *CAMTA1* could promote cyclin D1 expression. **(A)** CAMTA1 and cyclin D1 expressions in different SH-SY5Y cell lines. **(B)** Relative cell viabilities in different SH-SY5Y cell lines after incubation with OGD/R treatments (**P < 0.01). **(C)** Gentian Violet staining shows differences in cell proliferation after the treatment in different SH-SY5Y cell lines (**P < 0.01, ***P < 0.001). **(D)** CAMTA1 and cyclin D1 expressions in IS patients. **(E)** In HEK293T KO cell, the effect of CAMTA1 on *CCND1* transcriptional activity was evaluated using a luciferase reporter assay. (Statistical analysis of 3–6 independent experiments under each condition is shown in the column chart, and the error bar indicates ± 1 SD. *p < 0.05). ns stand for not statistically different.

Next, the new cell line was generated in SH-SY5Y *CAMTA1* KO cells with si*CCND1* (Figure 5A). The result of the CCK-8 assay demonstrated that decreased expression of the cyclin D1 reduced cell viabilities compared to SH-SY5Y *CAMTA1* KO cells (Figure 5B). The crystal violet staining was consistent with the cell viability data (Figure 5C). The results illustrated that cyclin D1 regulates cell proliferation induced by decreased CAMTA1. We also checked the cyclin D1 expression in IS patients (Figure 5D). Their expression is markedly elevated in patients having lower CAMTA1 levels. The results suggested that CAMTA1 controlled the cell cycle by regulating cyclin D1 expression in IS. As CAMTA1 is a transcription activator, a dual luciferase reporter assay was generated to investigate whether CAMTA1 directly regulates cyclin D1 expression. In HEK293T cells, the Firefly/Renilla ratio was not significantly changed with different expressions of CAMTA1, compared to without CAMTA1 (Figure 5E; Supplementary Figure 2F). It demonstrated that CAMTA1 could not directly affect the *CCND1* promoter region. There may be an intermediary between them.

## Discussion

Emerging evidence indicates that DNA methylation plays a role in the pathogenesis of IS. In this context, we analyzed the genome methylation levels from the peripheral blood samples of IS patients and healthy controls using an 850K Bead Chip. Out of the 622 CpG sites showing differential methylation, 80.4% (502 sites) exhibited hypermethylation, and 19.6% (122 sites) indicated hypomethylation, which suggests sufficient differences in the DNA methylation between patients and controls. These different sites were traced to 278 genes. According to our GO analysis and KEGG pathway analysis, they are mainly involved in the following pathways and functions: adhesion of cell membrane, adhesion molecules to homophilic cells, cell adhesion, phospholipid transport, neurogenesis regulation, calcium ion binding, collagen-binding, and sugar metabolism. All these pathways and functions have been previously reported to be related to the ischemic stroke (Love, 2003; Wen et al., 2005; Zündorf and Reiser, 2011; Zhao et al., 2020).

A disadvantage of our experience is the sample size (*n* = 3); we verified the top 19 differentially methylated genes: *ABCA1, ADAMTSL5, COL9A2, ERCC5, TGFBI, ABCG1, ATP104, CYP2EI, HOX44, PCDHB7, ARL4C, KLFI1, SULF2, ADARB2, GDFI5, CDHI5, CAMTAI, SMG6*, and *RNF144b*. Several DNA methylation modifications in the pathogenesis of IS have been investigated in the past few years. For example, the study in the middle cerebral artery occlusion (MCAO) in a rat model showed DNA methylation level of Na-K-Cl cotransporter 1 (NKCC1) was decreased (Lagarde et al., 2015). Hu et al. found the hypermethylation of thrombospondin 1 (THBS1), an angiostatic factor implicated in platelet aggregation, in an *in vitro* model of stroke. IS patients presented high plasma homocysteine levels associated with DNA hypermethylation of the thrombomodulin (TM) (Stanzione et al., 2020). Hypermethylation of cyclin-dependent kinase inhibitor 2B (CDKN2B), a gene involved in the pathogenesis of calcification, was also demonstrated to relate to calcification of the arteries in patients with IS (Unzeta et al., 2021). Baccarelli et al. suggested that the association between the hypomethylation of long interspersed nucleotide elements (LINE-1) and vascular cell adhesion protein 1 (VCAM-1) expression could be an early event in the etiology of cerebrovascular diseases, including IS (Lee et al., 2010). All these 19 genes have never been reported in the IS field. Although they could be new candidate genes related to the occurrence and development of stroke, we cannot rule out the possibility that this is just one bias from our experiment. Even though blood is commonly used in epigenomic studies, its heterogeneous nature leads to interpretation difficulties. For future research on defining their specific role in IS, we could verify their differential methylation levels from a selected single cell type, for example, lymphocytes.

We took CAMTA1 as the first candidate to be explored because its methylation markedly differs between patients and controls. Recently, Shen et al. also identified methylation modification of the *CAMTA1* gene in more than 400 IS patients (Shen et al., 2019). Furthermore, our MetylTarget analysis found that the methylation levels of CpG16 and CpG21 at the two sites in the IS group were significantly higher than those in the healthy control. It suggests that the hypermethylation of the *CAMTA1* gene promoter might relate to ischemic stroke.

*CAMTA1* gene was identified in 2003 as a candidate for tumor suppressor in neuroblastoma (Henrich et al., 2006). The following year, Nakatani et al. investigated the relationship between *CAMTA1* expression and cell cycle progression in N-type neuroblastoma SK-N-SH cells. They suggested that CAMTA1 could play a role in cell cycle regulation. Many studies examined its functions in various tumor cells, including breast cancer, colon cancer, pheochromocytoma, neuroblastoma, and glioma. The results showed that CAMTA1 could regulate tumor proliferation as an antitumor gene (Katoh and Katoh, 2003; Kim et al., 2006; Baccarelli et al., 2010a; Juhlin et al., 2015; Lu et al., 2018). Genetic studies of the WWTR1 (a protein known as TAZ)-CAMTA1 were well established in epithelioid hemangioendothelioma (EHE), a malignant vascular cancer. This discovery led *WWTR1-CAMTA1* fusions to become useful diagnostic markers for EHE (He et al., 2021). The mechanistic basis of the oncogenic functions of the TAZ-CAMTA1(TC) fusion protein has been distinctly defined. The fusion of CAMTA1 drove the constitutive nuclear localization to TAZ, and they escaped from the Hippo pathway regulation, rendering it constitutively active (Asgharzadeh et al., 2012).

Consequently, cells expressing TC oncoprotein display a TAZ-like transcriptional program that causes resistance to oncogenic transformation (Phurailatpam et al., 2015). More recently, He et al. demonstrated that CAMTA1 could regulate proliferation and the cell cycle in glioma by inhibiting AKT phosphorylation (He et al., 2021). Nevertheless, the function of CAMTA1 in neurological diseases is largely unknown. Just a few studies reported that the CAMTA1 gene had been associated with neonatal neuroblastoma, ataxia (Phurailatpam et al., 2015), and sporadic amyotrophic lateral sclerosis (Liang et al., 2020).

Our research focused on studying the function of CAMTA1 in strokes. The results showed that knockout of the *CAMTA1* gene in SH-SY5Y cells increased the proliferation and reduced the apoptosis after oxygen-glucose deprivation/reoxygenation, and more cells entered the S phase. The results revealed that the cell cycle was dysregulated in CAMTA1 KO cells. Our findings were consistent with the previous studies on tumor cells. CAMTA1 also plays an essential role in cell cycle regulation in strokes. We examined the changes of the mRNA and protein expression under CAMTA1 deletion to clarify its mechanisms in strokes. The *CCND1* mRNA and its protein cyclin D1 expression were significantly increased in *CAMTA1* KO cells. The functions of cyclin D1 are known for regulating the cell cycle's progression through the G1 to S phase (Fu et al., 2004). Increasing evidence demonstrates that dysregulation of cell cycle machinery is implicated in strokes. Significantly, many studies reported that cyclin D1 levels are increased in models of cerebral ischemia (Cai et al., 2009; Baccarelli et al., 2010b; Zhou et al., 2016). Our data suggest CAMTA1 could implicate IS through increasing cyclin D1. As we know, cyclin D1 could be regulated by different signal pathways, like the Hippo pathway, Jak/Stat pathway, etc. Our results established for the first time the link between CAMTA1 and cyclin D1. However, the dual-luciferase assay showed that there might not be a direct link between them. It is supposed that CAMTA1 could affect one intermediary protein that induces the cyclin D1 expression. *CCND1* gene is a downstream transcriptional target of the Hippo pathway, which was affected in SH-SY5Y CAMTA1 KO cells. One possibility is that a specific protein implicated in the Hippo signaling pathways could be regulated by CAMTA1. One limitation in our work is that we used SH-SY5Y cells, which is broadly studied for elucidating molecular mechanism in IS pathogenesis field. It's a neuroblastoma cell line, and we should explore the effect of CAMTA1 in a mouse model. CAMTA1 is principally expressed in the brain. For future study, we could decrease the expression of CAMTA1 in mouse brain and then study its effect under ischemic stress conditions.

In conclusion, (1) our study helped identify new candidate genes for the pathogenesis of IS. We found 19 genes with significant DNA methylation modifications in IS patients. All of them are involved in the pathways related to stroke. However, the sample size is our limitation. In future studies, this result should be validated using a large-scale sample size and selecting a single cell type (to avoid the bias due to the heterogeneous compositions from blood samples), and then define their specific functions in strokes. (2) Out of 19 genes, we concentrated on studying the role of CAMTA1 in Stroke because of its most different DNA methylation levels between healthy people and IS patients. Cell experiences demonstrated that CAMTA1 could affect cell proliferation and cell cycle in normal conditions or in the OGD/R model. (3) Moreover, decreased CAMTA1 could raise cyclin D1 levels. Our results showed for the first time that CAMTA1 plays a role in strokes by regulating cyclin D1, which increased under ischemic stress conditions. However, the mechanism by which CAMTA1 regulates cyclin D1 is presently unclear. As our result shows, there is no direct link between them; identifying their intermediary using a different neuron cell line and in vivo models could form part of future studies.

## Data availability statement

The datasets presented in this study can be found in online repositories. The names of the repository/repositories and accession number(s) can be found below: https://www.ncbi.nlm.nih.gov/geo/ GSE197080, GSE197081, and GSE205687.

## Ethics statement

The studies involving human participants were reviewed and approved by the Institutional Ethics Board of The First Affiliated Hospital of the Henan University of Chinese Medicine. The patients/participants provided their written informed consent to participate in this study.

## Author contributions

HZ and YHe designed the experiments. YLi and GS carried out most of the experiments. JJ, XZ, and FL analyzed the experimental results. CZ and YLi wrote the manuscript. ZL, YX, ZZ, SY, BZ, YLu, YHu, YP, and TH participated in the discussion of this project. All authors contributed to the article and approved the submitted version.

## Funding

This study was supported by the National Natural Science Foundation of China (Nos. U2004114 and 81571154).

## Conflict of interest

The authors declare that the research was conducted in the absence of any commercial or financial relationships that could be construed as a potential conflict of interest.

## Publisher's note

All claims expressed in this article are solely those of the authors and do not necessarily represent those of their affiliated organizations, or those of the publisher, the editors and the reviewers. Any product that may be evaluated in this article, or claim that may be made by its manufacturer, is not guaranteed or endorsed by the publisher.
