# Supplementary material for: CAMTA1 gene affects the ischemia-reperfusion injury by regulating CCND1
Source: Front Cell Neurosci. 2022 Sep 9;16:868291. doi: 10.3389/fncel.2022.868291 (PMC9500443; doi:10.3389/fncel.2022.868291)
Supplement: Supplementary file 1 [file Data_Sheet_1.pdf]

Supplementary

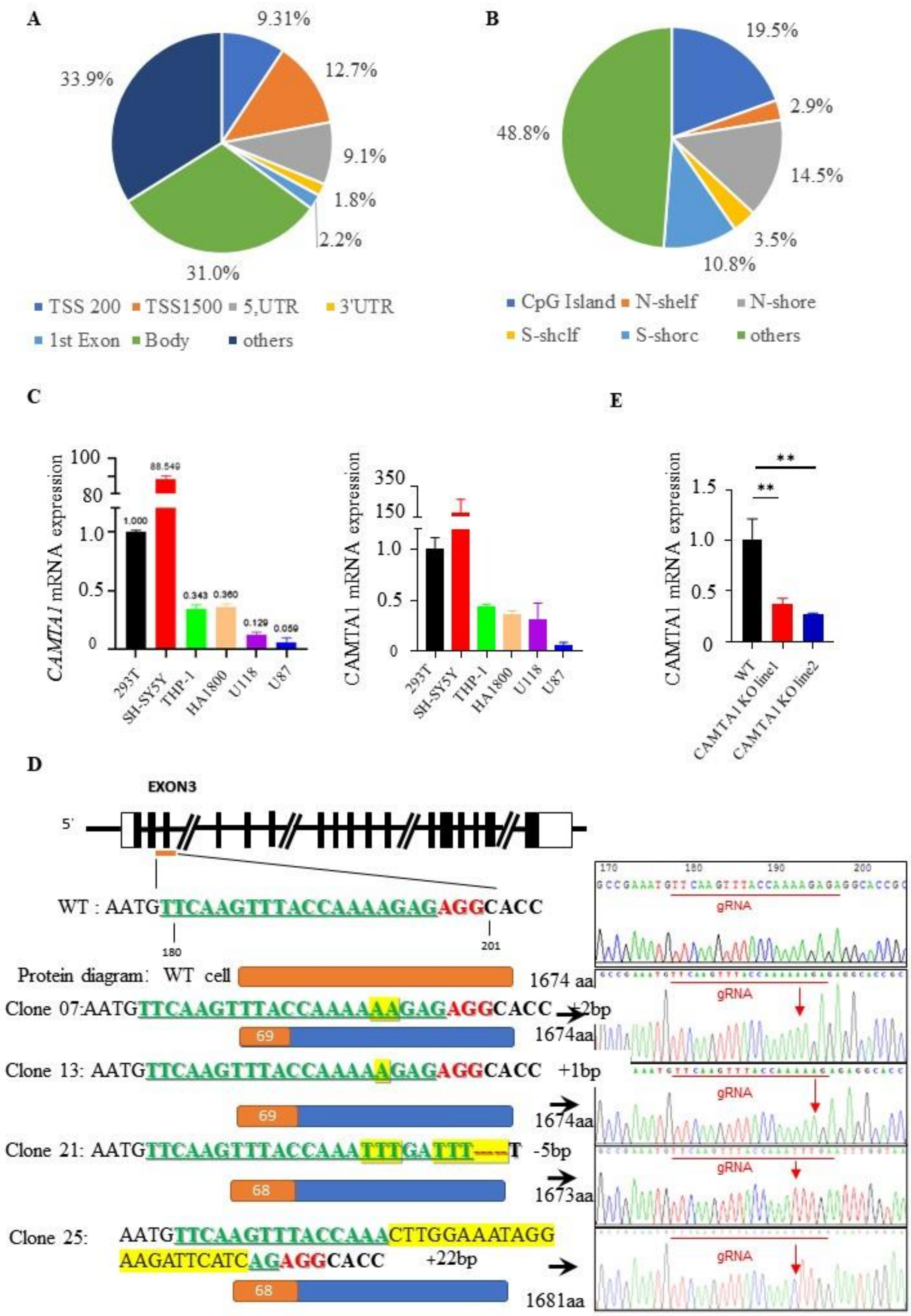

Supplementary Figure 1: A: The distribution of DMCs according to the site regions of the hypomethylation. B: The distribution of DMCs according to the genomic regions of the hypomethylation. C: mRNA expression of *CAMTA1* in different cell lines. D: Schematic diagram of *CAMTA1* gene KO by crispr/cas9. E: mRNA expression in WT SH-SY5Y cell lines and *CAMTA1* KO SH-SY5Y cell lines. (Statistical analysis of 3–6

independent experiments under each condition is shown in the column chart, and the error bar indicates ± 1 SD. *$p < 0.05$)

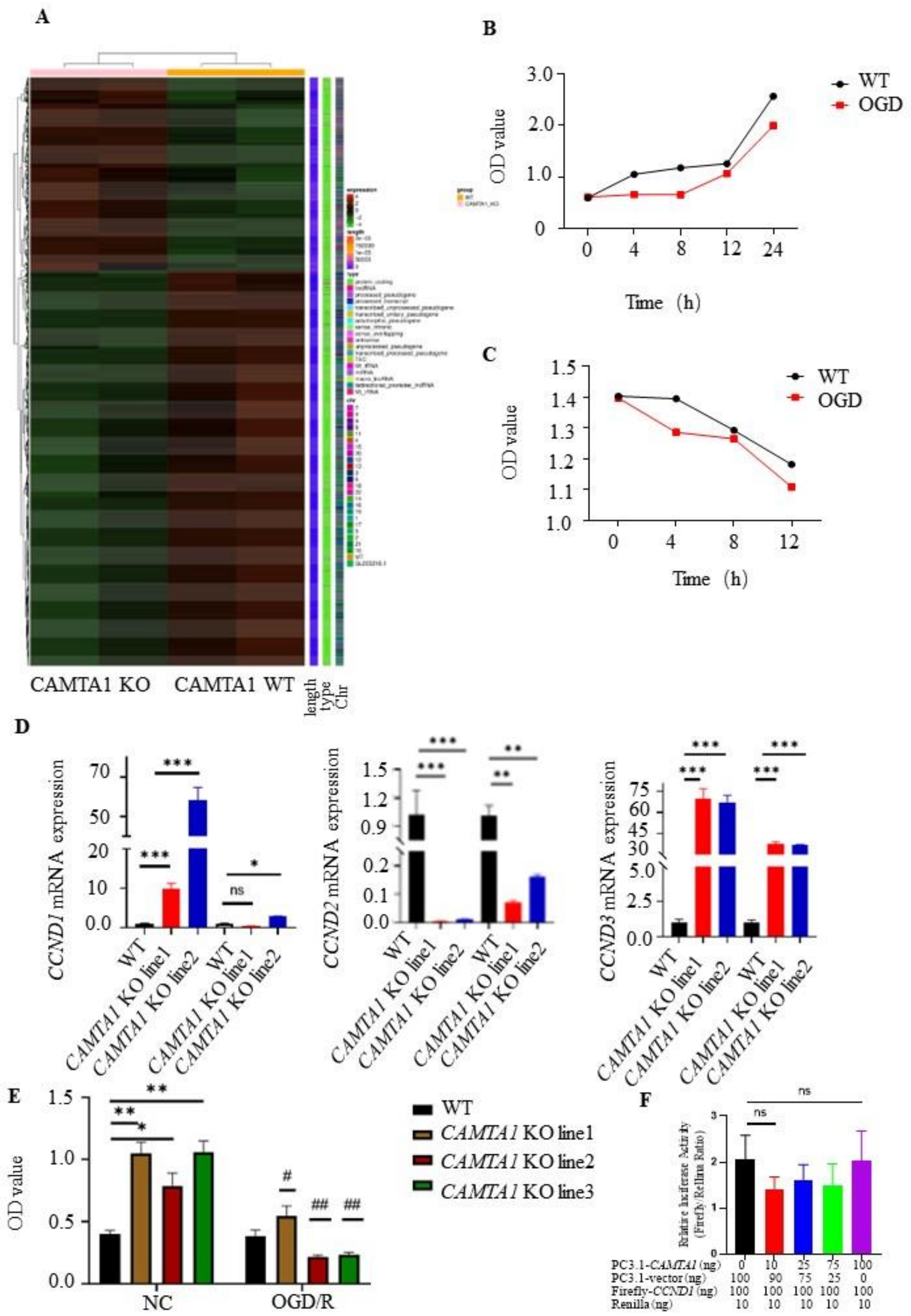

Supplementary Figure 2: A: heat map of 850K microarray. B-C: Cells were pretreated in anaerobic culture Incubator for 4 h and normal incubator for 4 hours, then colony formation was evaluated after two days (**P<0.01, ***P<0.001). D: mRNA expression of *CCND* families in WT SH-SY5Y cell lines and *CAMTA1* KO SH-SY5Y cell lines. E: Data are shown as percent cell viability standard deviation and normalized to the viability of *CAMTA1* KO and WT cell lines after OGD/R treatment. F: In HEK293T WT cells, the effect of CAMTA1 on *CCND1* transcriptional activity was

evaluated using a luciferase reporter assay. (Statistical analysis of 3–6 independent experiments under each condition is shown in the column chart, and the error bar indicates ± 1 SD. *p < 0.05)

Supplementary table 1: Characteristics of the two groups included in the 850K microarray analysis.

Types	IS（n=3）	Con（n=3）	t/χ2	*P*
Gender （male/female）	2/1	2/1	-	-
Age (years)	60.3±9.5	65.6±7.2	-0.986	0.38
Weight (kg)	62.5±7.3	68.6±8.0	0.976	0.385
MAP (mmHg)	95.1±13.7	102.3±15.2	0.609	0.575
BMI (kg/m2)	22.1±4.3	24.9±3.6	0.865	0.436
Totalcholesterol (mmol/L)	4.55±0.64	5.44±1.53	0.929	0.405
Bloodsugar (mmol/L)	6.930±2.58	6.383±2.89	0.245	0.819
Triglyceride (mmol/L)	2.281±0.791	3.128±0.719	1.372	0.242
Albumin (g/L)	41.63±4.73	39.38±5.53	0.530	0.621
HDL (mmol/L)	1.203±0.252	0.922±0.194	1.530	0.201
LDL (mmol/L)	2.134±0.960	3.211±0.883	1.43	0.226
HbA1c (%)	6.58±1.74	6.13±1.95	0.298	0.780
LVEF (%)	67.7±8.1	61.2±6.8	1.065	0.347
PLT (109/L)	180.5±61.7	262.7±140.3	0.926	0.407
MPV (fl)	8.80±1.82	7.92±1.21	0.697	0.524
PDW (fl)	16.78±0.80	16.57±0.52	0.381	0.722
ALT (U/L)	37.7±28.8	39.7±21.2	0.097	0.927
AST (U/L)	148.5±192.6	153.3±145.6	0.034	0.974
BUN (mmol/L)	5.456±0.886	5.995±0.935	0.725	0.509
Uricacid (μmmol/L)	277.2±133.7	263.3±80.9	0.154	0.885
Cr (μmol/L)	68.95±21.65	57.92±14.68	0.73	0.506

Supplementary table 2: Characteristics of the two groups included in the expanded population.

Types	IS（n=100）	Cons（n=100）	t/χ2	*P*
Gender (male/female）	55/45	58/42	0.183	0.669
Age (years)	61.3±8.5	67.4±7.8	0.083	0.934
Weight (kg)	69.5±7.6	67.6±8.5	1.82	0.07
MAP (mmHg)	96.3±12.8	102.3±16.6	2.858	0.005
BMI (kg/m2)	21.3±4.6	22.6±7.3	1.507	0.133
Totalcholesterol (mmol/L)	4.73±0.55	5.50±1.51	4.791	0.000
Bloodsugar (mmol/L)	6.59±2.58	6.17±2.36	1.201	0.231

Triglyceride (mmol/L)	2.881±0.531	3.958±0.459	15.334	0.000
Albumin (g/L)	40.63±4.73	39.38±5.53	1.718	0.087
HDL (mmol/L)	1.243±0.252	0.979±0.194	8.301	0.000
LDL (mmol/L)	2.689±0.570	3.156±0.669	5.313	0.000
HbA1c (%)	6.47±1.59	6.07±1.75	1.692	0.092
LVEF (%)	69.5±7.7	67.9±7.8	1.460	0.146
PLT (109/L)	180.5±61.7	212.7±140.3	2.101	0.037
MPV (fl)	8.00±1.82	7.88±1.21	0.549	0.584
PDW (fl)	16.90±0.56	16.82±0.30	1.806	0.072
ALT (U/L)	35.9±25.5	35.7±20.2	0.061	0.951
AST (U/L)	148.5±192.6	153.7±141.9	0.217	0.828
BUN (mmol/L)	5.864±0.348	5.795±0.345	1.408	0.106
Uricacid (μmmol/L)	268.8±1120.7	266.8±89.7	0.133	0.894
Cr (μmol/L)	66.13±18.90	69.35±14.63	1.347	0.179

Supplementary table 3: CpG site information of candidate genes

genes	CpG sites number	Lengths of CpG sites (bp)	Predict number of fragments.
ABCA1	2	654	2
ADAMTSL5	2	765	2
COL9A2	2	437	2
ERCC5	2	817	2
SMG6	1	971	I
TGFBI	1	620	1
ABCG1	2	869	2
ADARB2	2	2169	3
ATP10A	1	991	1
CYP2E1	2	1179	2
H0XA4	2	1058	2
PCDHB7	2	1216	2
ARL4C	1	1646	2
KLFU	3	2329	3
SULF2	3	1849	3
CDF15	1	921	2
CDH15	1	996	1
CAMTA1	2	2567	4
RNF144b	]	856	1

Supplementary table 4: The top 20 down-regulated KEGG pathways

Term_ID	Term_description	GeneSymbols	*P*_value
path:hsa04390	Hippo signaling pathway	BMP7;TEAD3;TP73;CTNNA2;SMAD3	0.014646
path:hsa04320	Dorso-ventral axis formation	SPIRE1;ETS2	0.028423
path:hsa05412	Arrhythmogenic right ventricular	ITGA2B;DES;CTNNA2	0.032884

	cardiomyopathy (ARVC)		
path:hsa00051	Fructose and mannose metabolism	AKR1B1;GMDS	0.038151
path:hsa00250	Alanine, aspartate and glutamate metabolism	DDO;GFPT2	0.044512
path:hsa00040	Pentose and glucuronate interconversions	AKR1B1;CRYL1	0.046714
path:hsa04014	Ras signaling pathway	FGF17;KSR2;NGFR;ETS2;ANGPT1	0.059817
path:hsa00520	Amino sugar and nucleotide sugar metabolism	GMDS;GFPT2	0.075972
path:hsa00561	Glycerolipid metabolism	AKR1B1;LPIN1	0.10659
path:hsa00790	Folate biosynthesis	ALPP	0.128618
path:hsa04015	Rap1 signaling pathway	FGF17;NGFR;ITGA2B;ANGPT1	0.132384
path:hsa04530	Tight junction	MYH2;EPB41L3;CTNNA2	0.137442
path:hsa04310	Wnt signaling pathway	DKK4;PRICKLE1;SMAD3	0.141651
path:hsa04514	Cell adhesion molecules (CAMs)	NCAM1;CD6;NEGR1	0.145903
path:hsa04520	Adherens junction	CTNNA2;SMAD3	0.149335
path:hsa05220	Chronic myeloid leukemia	RUNX1;SMAD3	0.149335
path:hsa05200	Pathways in cancer	PAX8;SMAD3;RUNX1;ITGA2B;CTNNA2;FGF17	0.161566
path:hsa04350	TGF-beta signaling pathway	BMP7;SMAD3	0.171839
path:hsa04146	Peroxisome	PEX5L;DDO	0.181653
path:hsa05410	Hypertrophic cardiomyopathy (HCM)	ITGA2B;DES	0.181653

Supplementary table 5: The top 20 up-regulated KEGG pathways

Term_ID	Term_description	GeneSymbols	*P*_value
path:hsa04514	Cell adhesion molecules (CAMs)	CDH15;HLA-B;HLA-C;CADM1	0.000838
path:hsa05330	Allograft rejection	HLA-B;HLA-C	0.005993
path:hsa05332	Graft-versus-host disease	HLA-B;HLA-C	0.00725
path:hsa04650	Natural killer cell mediated cytotoxicity	HLA-B;HLA-C;PRKCA	0.007627
path:hsa04940	Type I diabetes mellitus	HLA-B;HLA-C	0.00792
path:hsa03420	Nucleotide excision repair	BIVM-ERCC5;ERCC5	0.00934
path:hsa04145	Phagosome	NCF4;HLA-C;HLA-B	0.010884
path:hsa05320	Autoimmune thyroid disease	HLA-B;HLA-C	0.011263
path:hsa05416	Viral myocarditis	HLA-B;HLA-C	0.013781
path:hsa00140	Steroid hormone biosynthesis	SRD5A2;CYP2E1	0.013781
path:hsa04612	Antigen processing and presentation	HLA-B;HLA-C	0.023179

path:hsa05032	Morphine addiction	PRKCA;GRK5	0.032008
path:hsa04972	Pancreatic secretion	PRKCA;PRSS3	0.034556
path:hsa04144	Endocytosis	HLA-B;HLA-C;GRK5	0.041952
path:hsa04080	Neuroactive ligand-receptor interaction	TAAR2;PRSS3;TAAR5	0.049098
path:hsa04670	Leukocyte transendothelial migration	NCF4;PRKCA	0.049901
path:hsa04360	Axon guidance	EPHA8;NTN1	0.056778
path:hsa04380	Osteoclast differentiation	NCF4;FHL2	0.059937
path:hsa00591	Linoleic acid metabolism	CYP2E1	0.086732
path:hsa05164	Influenza A	PRKCA;PRSS3	0.098332

Supplementary table 6: Cellular Component results of the GO analysis

Term_ID	Term_description	ListHit	P_value
GO:0031901	early endosome membrane	8	0.000179
GO:0042612	MHC class I protein complex	3	0.00088
GO:0030054	cell junction	15	0.00126
GO:0005886	plasma membrane	74	0.001807
GO:0030673	axolemma	3	0.001892
GO:0097481	neuronal postsynaptic density	5	0.002981
GO:0009986	cell surface	16	0.00342
GO:0031966	mitochondrial membrane	5	0.004018
GO:0043025	neuronal cell body	11	0.004219
GO:0031430	M band	3	0.004412
GO:0070852	cell body fiber	2	0.008154
GO:0005858	axonemal dynein complex	2	0.011741
GO:0031225	anchored component of membrane	5	0.023625
GO:0005578	proteinaceous extracellular matrix	8	0.024328
GO:0005604	basement membrane	4	0.024869
GO:0032982	myosin filament	2	0.02576
GO:0002080	acrosomal membrane	2	0.02576
GO:0030286	dynein complex	2	0.02576
GO:0045211	postsynaptic membrane	7	0.027427
GO:0005911	cell-cell junction	6	0.028797

Supplementary table 7: Biological Process results of the GO analysis

Term_ID	Term_description	ListHit	*P*_value
GO:0007156	homophilic cell adhesion via plasma membrane adhesion molecules	15	2.13E-08
GO:0007155	cell adhesion	17	0.000265
GO:0045332	phospholipid translocation	4	0.000315
GO:0008152	metabolic process	16	0.001341
GO:0007411	axon guidance	14	0.001747
GO:0007417	central nervous system development	7	0.001904

GO:0016337	single organismal cell-cell adhesion	6	0.00346
GO:0060348	bone development	4	0.003497
GO:0050767	regulation of neurogenesis	3	0.003853
GO:0002474	antigen processing and presentation of peptide antigen via MHC class I	6	0.004399
GO:0001501	skeletal system development	7	0.005446
GO:0030853	negative regulation of granulocyte differentiation	2	0.007139
GO:0001539	cilium or flagellum-dependent cell motility	2	0.007139
GO:0010529	negative regulation of transposition	2	0.007139
GO:0009966	regulation of signal transduction	3	0.008482
GO:0007275	multicellular organismal development	13	0.010566
GO:0002480	antigen processing and presentation of exogenous peptide antigen via MHC class I, TAP-independent	2	0.010699
GO:0006497	protein lipidation	2	0.010699
GO:0060395	SMAD protein signal transduction	4	0.012066

Supplementary table 8 Molecular Function results of the GO analysis

Term_ID	Term_description	ListHit	*P*_value
GO:0005509	calcium ion binding	22	0.000327
GO:0004012	phospholipid-translocating ATPase activity	3	0.001889
GO:0005518	collagen binding	5	0.002145
GO:0000978	RNA polymerase II core promoter proximal region sequence-specific DNA binding	11	0.010942
GO:0042605	peptide antigen binding	3	0.011758
GO:0044212	transcription regulatory region DNA binding	7	0.015077
GO:0005251	delayed rectifier potassium channel activity	3	0.017067
GO:0003700	transcription factor activity, sequence-specific DNA binding	20	0.017194
GO:0003777	microtubule motor activity	4	0.017489
GO:0008375	acetylglucosaminyltransferase activity	2	0.020561
GO:0035326	enhancer binding	2	0.020561
GO:0005160	transforming growth factor beta receptor binding	3	0.020795
GO:0000287	magnesium ion binding	7	0.021087

GO:0001228	transcriptional activator activity, RNA polymerase II transcription regulatory region sequence-specific binding	4	0.022841
GO:0031267	small GTPase binding	2	0.028508
GO:0005543	phospholipid binding	4	0.032561
GO:0008092	cytoskeletal protein binding	3	0.032745
GO:0050840	extracellular matrix binding	2	0.034378
GO:0017137	Rab GTPase binding	4	0.036262

Supplementary table 9: RNA seq results in different SH-SY5Y cell lines

**Gene_ID**	***P*_value**	***P*_adj**	**Gene_Name**
ENSG00000156508	1.54E-09	4.21E-08	EEF1A1
ENSG00000167658	4.15E-17	3.95E-15	EEF2
ENSG00000140988	4.86E-16	3.83E-14	RPS2
ENSG00000198804	0.000712989	0.00438432	MT-CO1
ENSG00000184009	0.614865076	0.778144283	ACTG1
ENSG00000075624	0.814268207	0.904601964	ACTB
ENSG00000089157	1.00E-14	6.58E-13	RPLP0
ENSG00000198938	3.65E-06	4.34E-05	MT-CO3
ENSG00000161016	2.67E-18	2.91E-16	RPL8
ENSG00000198712	0.002934763	0.014373833	MT-CO2
ENSG00000074800	4.05E-17	3.88E-15	ENO1
ENSG00000168028	2.20E-39	1.40E-36	RPSA
ENSG00000166165	1.36E-38	8.03E-36	CKB
ENSG00000167526	2.32E-20	3.36E-18	RPL13
ENSG00000174444	1.16E-16	1.01E-14	RPL4
ENSG00000096384	9.60E-08	1.69E-06	HSP90AB1
ENSG00000100316	2.05E-19	2.63E-17	RPL3
ENSG00000169710	1.24E-28	4.02E-26	FASN
ENSG00000198899	0.000108325	0.000857759	MT-ATP6
ENSG00000067225	3.02E-11	1.09E-09	PKM

ENSG00000204628	1.94E-16	1.61E-14	RACK1
ENSG00000111640	5.50E-22	9.73E-20	GAPDH
ENSG00000143799	1.70E-06	2.24E-05	PARP1
ENSG00000142541	7.70E-20	1.06E-17	RPL13A
ENSG00000240342	1.26E-13	6.71E-12	RPS2P5
ENSG00000198034	8.81E-11	2.94E-09	RPS4X
ENSG00000196230	9.58E-11	3.17E-09	TUBB
ENSG00000198886	0.036104132	0.109004467	MT-ND4
ENSG00000109971	0.008686334	0.034825772	HSPA8

P_value means CAMTA1 KO SH-SY5Y cell lines Vs WT SH-SY5Y cell lines
